# S100A12 concentrations and myeloperoxidase activity in the intestinal mucosa of healthy dogs

**DOI:** 10.1186/s12917-015-0551-1

**Published:** 2015-09-14

**Authors:** Mohsen Hanifeh, Romy M. Heilmann, Satu Sankari, Minna M. Rajamäki, Laura Mäkitalo, Pernilla Syrjä, Susanne Kilpinen, Jan S. Suchodolski, Jörg M. Steiner, Thomas Spillmann

**Affiliations:** Department of Equine and Small Animal Medicine, Faculty of Veterinary Medicine, University of Helsinki, PO Box 57, Viikintie 49, 00014 Helsinki, Finland; Department of Clinical Sciences, Faculty of Veterinary Medicine, University of Tabriz, Postal code 5166616471 Tabriz, Iran; Gastrointestinal Laboratory, Department of Small Animal Clinical Sciences, College of Veterinary Medicine and Biomedical Sciences, Texas A&M University, College Station, TX 77843-4474 USA; Children’s Hospital, Helsinki University Central Hospital, University of Helsinki, PO Box 63, Haartmaninkatu 8, 00014 Helsinki, Finland; Department of Veterinary Biosciences, Faculty of Veterinary Medicine, University of Helsinki, PO Box 66, Agnes Sjöberginkatu 2, 00014 Helsinki, Finland

**Keywords:** S100A12, Myeloperoxidase, Intestine, Dog

## Abstract

**Background:**

Relatively few laboratory markers have been evaluated for the detection or monitoring of intestinal inflammation in canine chronic enteropathies, including inflammatory bowel disease (IBD). Previous research found that the intestinal mucosal levels of S100A12 and myeloperoxidase (MPO), as biomarkers of gut inflammation, were elevated in human patients with IBD. To date, the S100A12 and MPO levels in intestinal mucosal samples from either healthy dogs or from dogs suffering from IBD remain unreported. Therefore, this study aimed to evaluate the mucosal S100A12 and MPO levels in four different parts of the intestine (duodenum, jejunum, ileum and colon) in 12 healthy laboratory Beagle dogs using the ELISA and spectrophotometric methods, respectively.

**Results:**

Based on histological examinations, the recorded findings for all the samples were considered normal. The mucosal concentration of S100A12 in the ileum was significantly higher than in all other segments of the intestine (*p* < 0.05). MPO activity was significantly higher in the ileal, jejunal and duodenal than in colonic mucosal samples (*p* < 0.05). Moreover, its concentration was higher in the jejunum than in the duodenum.

**Conclusions:**

This study showed that S100A12 and MPO are reliably detectable in canine intestinal mucosa. The assays used appeared to be sufficient to further evaluate the role of S100A12 and MPO in the pathogenesis of canine chronic enteropathies, including IBD. These biomarkers may play a role in the initial detection of gut inflammation suggesting the need for further investigations to confirm IBD or to differentiate between IBD subtypes. Understanding the role of S100A12 and MPO in the pathogenesis of chronic intestinal inflammation in future may result in an improved understanding of canine chronic intestinal inflammation.

## Background

In canine medicine, a limited number of laboratory markers for the detection or monitoring of localised (e.g., gastrointestinal) or systemic inflammation exists [1]. The diagnosis of canine inflammatory bowel disease (IBD), for example, often poses challenges for the veterinarian and relies on the exclusion of other diagnoses and the histopathological evaluation of intestinal mucosal biopsies [2, 3]. In addition, neither clinical signs nor clinicopathological markers allow the clinician to predict whether a dog with chronic idiopathic IBD will respond to a change in diet alone, to antibiotics or to corticosteroids [4]. Thus, objective biomarkers may help to simplify diagnosis and possibly predict the most appropriate treatment approach in canine IBD.

S100A12 (also known as calgranulin C) belongs to the S100/calgranulin-protein family and represents a useful inflammatory marker in human patients with inflammatory diseases, such as systemic lupus erythematosus [5], rheumatoid arthritis [6], respiratory disease [7, 8] and IBD [9–16]. S100A12 is mostly expressed and secreted by activated phagocytes [17] and, at lower levels, by keratinocytes [18], monocytes [19] and eosinophils [19]. S100A12 plays a role in intracellular homeostasis and possesses extracellular functions, including chemotaxis, proinflammatory cytokine production, sustained recruitment of leukocytes and induction of oxidative stress [19–21]. Increased S100A12 concentrations were found in the stool samples, serum and intestinal mucosa in human patients with IBD [10–16]. Recently, canine S100A12 was purified [22], and a radioimmunoassay was developed and analytically validated for its quantification in serum and faecal [23] and urine samples from dogs [24]. Furthermore, an S100A12 concentration was recently measured as a non-invasive marker in the faeces of dogs with and without IBD using the ELISA method [25]. That study showed that dogs with IBD have higher faecal S100A12 concentrations than healthy dogs. However, when measuring only the faecal S100A12 concentration, it is impossible to know from which part of the intestine they originate. Given the various roles of S100A12, it is reasonable to consider this protein’s function in the intestinal mucosa during inflammation.

Myeloperoxidase (MPO) is an enzyme found mostly in neutrophils as well as at lower concentrations in monocytes and macrophages [26, 27]. It plays a major role in intracellular microbial destruction, but, at inflammatory sites, it is released into the extracellular space after phagocyte activation and induces damage to the host tissue [28]. Increased intestinal tissue levels of MPO as a biomarker of oxidative stress were found in the animal models of IBD [29–31] and in both forms of human IBD [Crohn’s disease (CD) and ulcerative colitis (UC)] [32–34]. This finding raises the question of whether mucosal MPO activity may also be associated with canine IBD. In canine IBD, the predominant inflammatory cells are thought to be lymphocytes and plasma cells. However, German et al. also found a significant increase in the number of neutrophils and macrophages in dogs with antibiotic-responsive diarrhoea and in dogs with IBD when compared to healthy controls [35]. To our knowledge, no reports of S100A12 and MPO levels in intestinal mucosal samples from healthy dogs or dogs with IBD exist.

To initiate research on this aspect of canine intestinal inflammation, our study aimed to determine the mucosal S100A12 and MPO levels in different parts of the intestine in 12 healthy laboratory Beagle dogs. The results of this study provide the basis for future studies regarding the role of mucosal S100A12 and MPO as inflammatory biomarkers in the pathogenesis of canine chronic enteropathies, including IBD.

## Results

### Histological examination

Based on the histological examination of the intestine, the median total WSAVA score of all samples was 0 (range 0–3), classifying all findings as normal. We found no histological abnormalities in the submucosa, the muscularis externa or the serosa in any of the samples.

### S100A12 concentrations in the intestinal mucosa

**Fig. 1:**
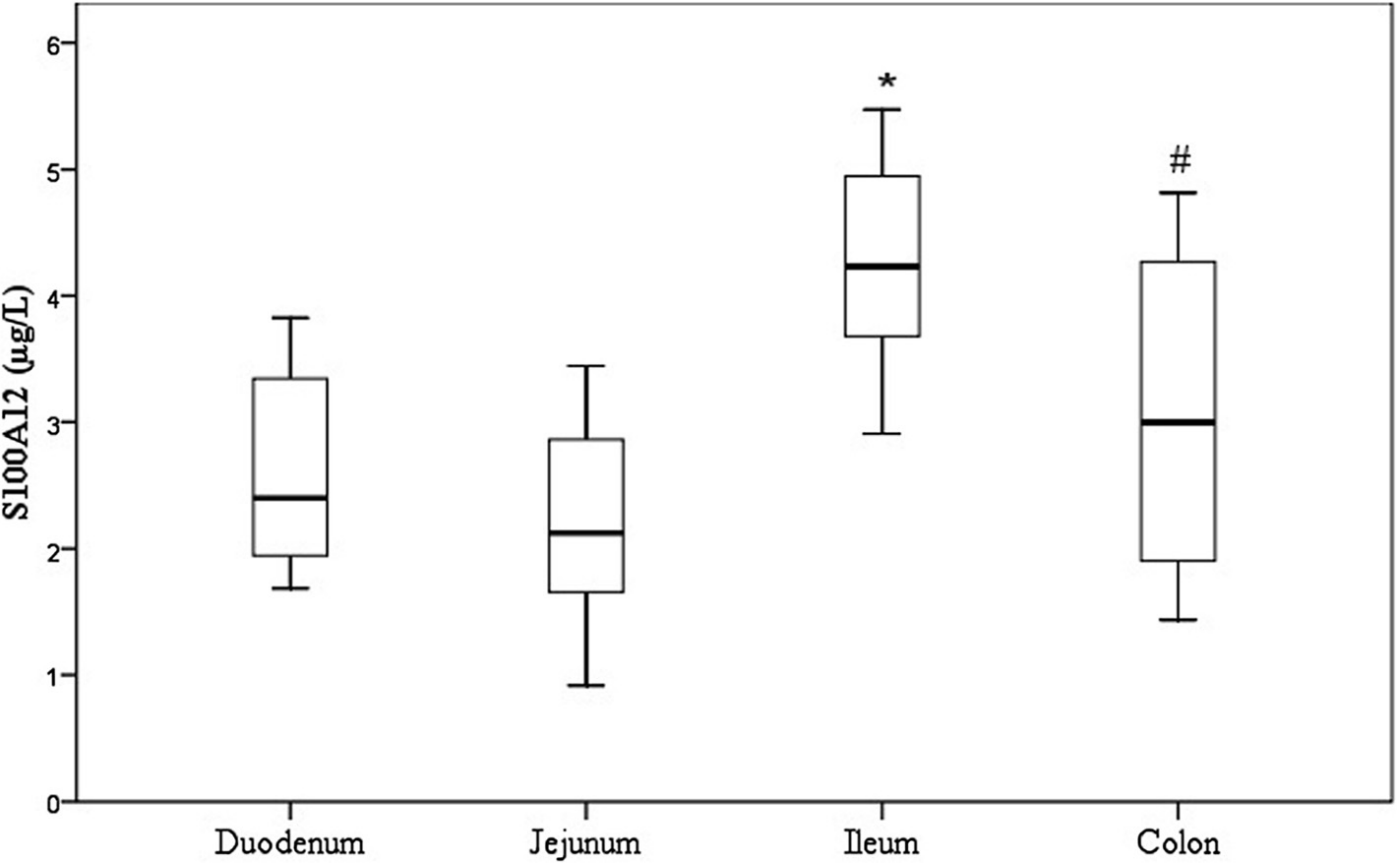
Boxplot representing the log-transformed S100A12 concentrations in the intestinal mucosal samples of 12 healthy dogs. The horizontal line inside each box represents the median; the top and bottom of each box represent the 75th and 25th percentiles, respectively; and the whiskers represent the 95th and 5th percentiles. ^*^ *p* < 0.05 vs. duodenum, jejunum and colon; # *p* < 0.05 vs. jejunum

The S100A12 concentration in all samples ranged from 2.5–237.6 μg/L. In the four different parts of the intestine examined in 12 healthy dogs, the highest median levels of S100A12, which were significant, were found in the ileum (71.5 μg/L [38.9–141.9]), followed by the colon (23.2 μg/L [6.7–75.6]), duodenum (11.4 μg/L [6.9–28.5]) and jejunum (8.5 μg/L [5.1–19.3]) (Table 1 and Fig. 1). In addition, the difference between the colonic and jejunal mucosa was significant (*p* < 0.05).

**Table 1:** Distribution of the S100A12 concentrations and MPO activities in the intestinal mucosal samples of 12 healthy dogs

Intestinal segment	Dog No	1	2	3	4	5	6	7	8	9	10	11	12
	Variable												
Duodenum	S100A12 (μg/L)	6.0	27.9	7.3	5.4	27.0	45.8	28.7	14.0	30.3	8.7	6.7	8.0
	MPO (ΔA/min)	0.61	0.35	0.46	0.03	0.17	0.09	0.7	0.08	0.11	0.62	0.54	0.1
Jejunum	S100A12 (μg/L)	7.3	22.1	2.5	5.0	31.3	20.8	9.6	5.5	11.9	5.8	2.7	14.8
	MPO (ΔA/min)	5.12	1.04	2.15	0.22	0.26	0.32	1.92	0.37	0.22	0.35	0.85	0.36
Ileum	S100A12 (μg/L)	38.1	143.0	41.2	18.3	91.6	209.6	138.4	90.8	237.6	52.1	44.1	20.0
	MPO (ΔA/min)	0.18	1.14	1.25	0.16	0.8	1.33	1.43	0.18	0.22	0.72	0.55	0.28
Colon	S100A12 (μg/L)	11.5	123.4	64.4	5.2	79.3	57.6	4.2	6.8	34.9	94.4	6.6	7.0
	MPO (ΔA/min)	0.2	0.12	0.14	0.06	0.1	0.14	0.03	0.05	0.04	0.52	0.03	0.09

### Analytical validation of the MPO method and MPO activity in the intestinal mucosa

The intra-assay coefficients of variation (CV %) in six different small intestinal mucosa samples assayed in a 1:2 dilution were 0.77, 0.85, 1.80, 1.56, 0.64 and 1.02 %, and were 1.55, 1.84, 1.24, 1.30, 1.03 and 1.03 % in six different large intestinal mucosa samples tested in a 1:2 dilution. The inter-assay CV % for six different small intestinal and six different large intestinal mucosa samples assayed in a 1:2 dilution were 1.77, 0.37, 2.55, 2.24, 1.32 and 2.59 %, and 7.00, 7.59, 1.76, 2.74, 1.00 and 1.66 %.

The observed to expected ratios (O/E) at dilutions of 1:2, 1:4, 1:8 and 1:16 ranged from 91.6–106.9 % (mean ± SD: 98.3 ± 3.8 %) for six different small intestinal mucosa samples, and from 84–103.8 % (93.6 ± 4.9 %) for six different large intestinal mucosa samples. O/E for the spiking recovery ranged from 97.3–111.1 % (103.7 ± 4.8 %) for five different small intestinal mucosa samples, and from 90.2–103.8 % (97.1 ± 4.4 %) for five different large intestinal mucosa samples.

**Fig. 2:**
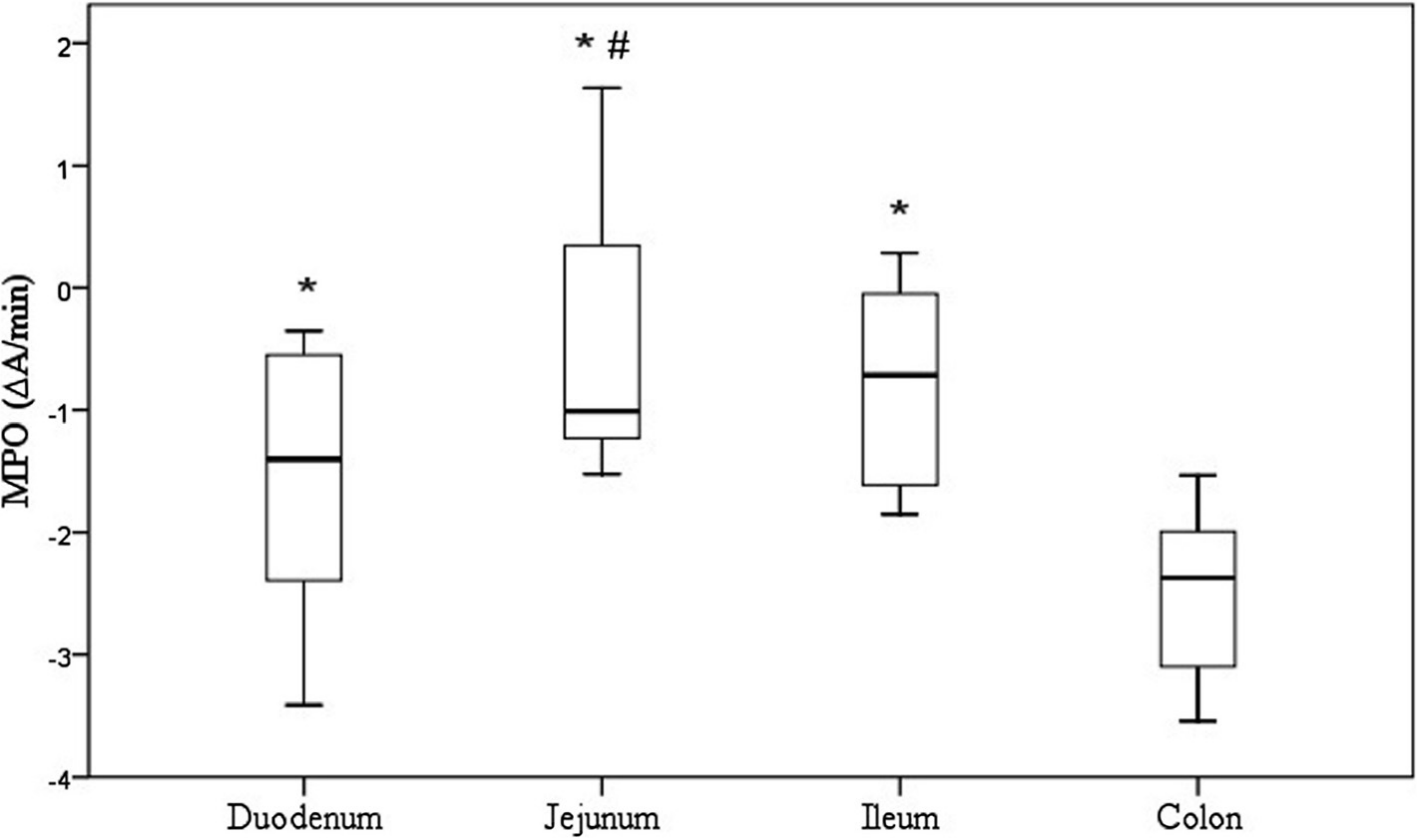
Boxplot representing the log-transformed MPO activities in the intestinal mucosal samples of 12 healthy dogs. The horizontal line inside each box represents the median; the top and bottom of each box represent the 75th and 25th percentiles, respectively; and the whiskers represent the 95th and 5th percentiles. ^*^ *p* < 0.05 vs. colon; # *p* < 0.05 vs. duodenum

The MPO enzyme activity was determined in all mucosal samples from four different intestinal areas of 12 healthy Beagles. The median MPO activity was significantly higher in the ileum (0.49 ΔA/min [0.19–1.05]), jejunum (0.36 ΔA/min [0.28–1.70]) and duodenum (0.26 ΔA/min [0.09–0.59]) than in the colon (0.09 ΔA/min [0.04–0.14]) (*p* < 0.05) (Table 1 and Fig. 2). In addition, the difference between the jejunal and duodenal mucosa was significant (*p* < 0.05) (Fig. 2).

## Discussion

This study aimed to determine the concentration of S100A12 and the activity of MPO in four different parts of the intestine in healthy Beagles by using ELISA and a spectrophotometric method, respectively. The ELISA method was validated as capable of determining S100A12 in the faecal samples of healthy dogs [25] and dogs with IBD [25], but to the best of our knowledge not in canine intestinal mucosal extracts. In this study, we successfully established and validated for the first time a spectrophotometric method to measure MPO activity in the intestinal mucosa in healthy dogs.

According to the literature, the interpretation of gastrointestinal histopathological findings may vary widely between pathologists even with standardised grading schemes [36, 37]. Despite using a pictorial template, Willard et al. found poor agreement between pathologists evaluating the number of duodenal neutrophils in dogs and cats when using a four-point scale (0 = normal, 1 = mild, 2 = moderate, 3 = severe) [37]. Hence, applying objective biomarkers may overcome this problem. S100A12 and MPO are exclusively expressed in relevant amounts by granulocytes, which play an important role in the pathogenesis of IBD in humans [15, 34].

Elevated intestinal mucosal concentrations of S100A12 were found in human patients with IBD. When secreted extracellularly, S100A12 contributes to innate immune responses and activates intracellular signalling cascades leading to cytokine production and the induction of oxidative stress in IBD patients [38]. In the present study, S100A12 concentrations were higher in extracts from the ileum than in other segments of the intestine. S100A12 detection in the intestinal mucosa may reflect the number of neutrophils infiltrating the mucosa. Because of poor agreement between pathologists evaluating the number of duodenal neutrophils [37], we did not attempt to perform neutrophil count comparisons between the different intestinal sections. Therefore, we assume that the higher S100A12 concentrations in the ileum may be due to the high number of S100A12-expressing cells (neutrophils and macrophages) within the ileal mucosa. Heilmann et al. reported variations in the faecal concentrations of S100A12 in healthy dogs [23]. An irregular distribution of S100A12-expressing cells within the gastrointestinal mucosa or variation in the concentration of faecal proteins due to variations in the gastrointestinal passage were suggested as possible reasons for this variation [23]. Moreover, Heilmann et al. reported a similar variation in other faecal markers in healthy dogs [39, 40]. In our study, the S100A12 concentration in all mucosal extracts ranged from 2.5–237.6 μg/L. Similar variations in the S100A12 concentration were reported for normal duodenal and cecal mucosal biopsies in humans [13]. In a study by Leach et al., the S100A12 concentration ranged from 7–36 μg/L in the culture supernatants of duodenal and cecal mucosal biopsies obtained from 33 non-IBD control children [13]. Duodenal and cecal biopsies were also cultured and supernatants were collected for the determination of S100A12 using the ELISA method. A similar variation was also found in the concentration of calprotectin, another member of the S100/calgranulin protein family, ranging from 16–511 μg/L in the culture supernatants of duodenal and cecal mucosal biopsies [13].

MPO is an enzyme found predominantly in neutrophils and in lower concentrations in monocytes and macrophages. The quantification of MPO is well established for the evaluation of intestinal inflammation in humans and animal models of IBD [32, 33, 41–43]. The number of neutrophils and consequently MPO enzyme activity increase in inflammatory conditions such as human and animal models of IBD [29, 32, 33, 41–43]. Previously, MPO-derived hypochlorous acid (HOCl) was shown to affect oxidative tissue damage in human IBD [34]. In our study, MPO activities were higher in the ileal, jejunal and duodenal mucosa than in the colonic mucosa of healthy dogs. Moreover, its concentration was higher in the jejunum than in the duodenum. The detection of MPO in the intestinal mucosa could reflect the number of granulocytes infiltrating the mucosa. Variations in the MPO activity in our study in different parts of the intestine in healthy dogs could be explained by the disparity in the distribution of MPO-releasing cells, such as the neutrophils and macrophages. Several studies investigated the distribution of immune cells in the intestines of healthy dogs and cats [43–47]. However, the results did not allow for conclusions concerning the distribution of MPO, since the distribution of neutrophils as the primary source of MPO was not studied. Macrophages, as a possible source of MPO, were detected using the antibody MAC387 in two studies of the intestines of normal cats [47] and dogs [43]. The authors concluded that the number of MAC387 positive cells was higher in the ileum than in other segments of the intestine. However, to our knowledge, no study addressing the distribution of the MPO enzyme in different parts of the normal intestinal mucosa of dogs or any other species exists.

Considering the lack of sufficiently sensitive and specific biomarkers for canine IBD, identifying such a marker would substantially improve diagnosis and disease monitoring, and may potentially lead to a significant improvement in patient care and quality of life. Faecal and serum calprotectin, as a non-invasive biomarker, was found to increase in dogs with IBD [48, 49]. In another study, faecal concentrations of calprotectin were significantly higher in dogs with chronic diarrhoea than in healthy control dogs [50]. Furthermore, faecal S100A12 concentrations were higher in dogs with IBD [25]. Whilst using non-invasive biomarkers is more appropriate in determining the presence of gut inflammation, it is impossible to correlate faecal S100A12 with certain histological subtypes of IBD (lymophocytic-plasmacytic enteritis, eosinophilic enteritis, etc.), since the specific type causing an increase and to what degree remain unknown. In addition, measuring the faecal S100A12 concentration does not allow for the assessment of the specific part of the intestine from which the S100A12 sample originates. In our opinion, knowing which part of the intestine has the highest level of S100A12 is important as a possible means for differentiating canine IBD subtypes*.* Faecal S100A12 may be valuable for screening dogs suspected of having IBD and for monitoring disease activity, thus reducing the need for endoscopy during disease follow-up. Increases in faecal S100A12 are, therefore, viewed as indicative of endoscopy, rather than being used as an alternative to endoscopy for diagnosis [10]. In our study, we validated laboratory methods to determine the biomarkers in tissues from their proposed origin. Both mucosal S100A12 and MPO may be valuable markers complementing invasive diagnostic measures when used in combination with intestinal mucosal histology. This is an important basis for the study of the role of such markers in different types of canine IBD. To our knowledge, studies determining S100A12 and MPO in intestinal mucosal samples from dogs suffering from IBD remain unreported.

Extensive descriptions exist of the presence of increased levels of both S100A12 and MPO in IBD patients in humans [10–16, 32, 33, 51, 52]. Ileal and colonic mucosal S100A12 concentrations distinguished active IBD human subjects from healthy controls with a high sensitivity and specificity [15]. In another study, S100A12 concentrations in duodenal and cecal mucosal samples were significantly higher in children with IBD compared with non-IBD controls [13]. Foell et al. found a significant correlation between ileal and colonic mucosal S100A12 concentrations and intestinal tissue inflammation [15]. In addition, a significant correlation was also found between faecal and serum S100A12 concentrations and disease activity index in adults [11, 16] with CD and UC. Therefore, some researchers concluded that S100A12 is a suitable non-invasive marker for monitoring disease activity [11, 16] and predicting disease relapse both in CD and UC [16].

Studies of the correlation between S100A12 and the paediatric Crohn’s disease activity index (PCDAI) in children presented conflicting results. Whilst most studies report no correlation [13, 14, 16], De Jong et al. found a significant correlation in children with Crohn’s disease [10]. Heilmann et al. reported similar findings in dogs with IBD, finding a significant correlation between faecal S100A12 and the canine chronic enteropathy clinical activity index (CCECAI) [25]. Such studies need, however, to take into consideration that both PCDAI and CCECAI include a number of subjective elements, and consequently do not always represent the actual inflammatory burden, possibly leading to disagreements between results from different studies.

Faecal myeloperoxidase levels were also significantly correlated with histological indices of disease activity in UC [51]. In addition, Wagner et al. showed that normalised faecal MPO levels could be used to predict a complete clinical response to treatment among patients with CD or UC [52].

Whether S100A12 and MPO are also involved in the pathogenesis of canine IBD remains unknown and requires further investigation. In the current study, we aimed to provide basic data for the determination of S100A12 concentrations and MPO activities in the intestinal mucosa of healthy dogs. Our study forms the basis for on-going clinical trials comparing S100A12 and MPO in subtypes of IBD. In future studies, immunohistochemistry should also be included to assess the localisation of S100A12 and MPO in canine intestinal mucosa and their association with intestinal pathologies in dogs.

## Conclusions

In conclusion, we determined the concentrations of S100A12 and MPO in intestinal mucosa samples from healthy dogs in this study, laying the groundwork for future studies in canine chronic intestinal diseases, including IBD. Both mucosal biomarkers may play a role in the initial detection of gut inflammation leading to further investigations confirming IBD or to differentiating between IBD subtypes. Understanding the role of S100A12 and MPO in the pathogenesis of chronic intestinal inflammation in future and evaluating their use as biomarkers to distinguish between dogs with idiopathic IBD versus healthy dogs or dogs with food-responsive diarrhoea, antimicrobial-responsive diarrhoea and other gastrointestinal diseases may provide the possibility of improving our understanding of canine chronic intestinal inflammation and the current treatment approach.

## Methods

### Sample collection and processing

We obtained intestinal tissue samples from 12 healthy laboratory Beagle dogs (8 males, 4 females; median age: 11 years, range: 10–13 years) during post-mortem examinations upon completion of another unrelated study, which was approved by the Finnish National Animal Experiment Board (study license No. ESLH-2007-09833/Ym-23). The dogs were housed according to the European Union guidelines in groups in indoor pens with access to outdoor runs. The indoor environmental temperature was maintained at between 15–24 °C. The dogs were exposed to both natural and artificial light (from 7:00–16:00). They were fed a standard commercial diet and were evaluated as healthy based on history, physical examination, complete blood count, serum biochemistry and faecal examination. Immediately after humane euthanasia, their intestinal tracts were opened longitudinally and flushed with cold saline. Full-thickness tissue samples were collected from four different segments of the intestine (duodenum, jejunum, ileum and colon), snap-frozen in liquid nitrogen and stored at −80 °C for S100A12 and MPO determination. For histological evaluation, parts of the frozen intestinal tissue samples were later slowly thawed and fixed in 4 % formaldehyde solution in phosphate buffered saline (PBS) at 8 °C under permanent automatic rotation of the sample tube. Then, the samples were trimmed and embedded in paraffin wax. Sections (3–5 μm) were prepared and stained with haematoxylin and eosin for histological examination.

### Assessment of intestinal health

All dogs were healthy without any signs of gastrointestinal diseases based on their histories, physical examinations and laboratory examinations of their faeces and blood. Histological assessment of the intestinal samples was performed using the guidelines of the World Small Animal Veterinary Association (WSAVA) Gastrointestinal Standardisation Group [53]. In all small bowel samples, five morphological features (villous stunting, epithelial injury, crypt distension, lacteal dilation and mucosal fibrosis) and three types of infiltrated leukocytes (intraepithelial lymphocytes, lamina propria lymphocytes and lamina propria neutrophils) were selected and scored from 0 to 3 according to the WSAVA standardisation guidelines. In the colonic samples, we also evaluated the crypt hyperplasia, dilatation and distortion. The total score was classified as normal (score 0–4), mild (score 5–9), moderate (score 10–14), severe (score 15–19) or very severe (score ≥ 20).

### Measurement of mucosal S100A12 concentration

Snap-frozen intestinal mucosal samples (50 mg) from the duodenum, jejunum, ileum and colon were homogenised for 2 × 50 s at 5000 × g in 1 mL of ice-cold extraction buffer containing 50 mM Tris/HCl base, 150 mM NaCl, 10 mM CaCl_2_, 0.2 mM NaN_3_ and 0.01 % (*v/v*) Triton X-100 (pH 7.6) in the presence of EDTA-free protease inhibitor cocktail tablets (Complete EDTA-free tablets, Roche, Basel, Switzerland) using Precellys 24 ceramic beads at 4 °C (Bertin technologies, Paris, France). After homogenisation, samples were centrifuged at 13 000 × g and 4 °C for 10 min, and the supernatants were collected and stored at −80 °C for the measurement of S100A12. The S100A12 concentration was determined in intestinal mucosal samples obtained from four different intestinal segments from 12 healthy laboratory Beagle dogs using the ELISA method, which was recently developed and validated [25]. Briefly, immunoassay plates were coated with affinity-purified polyclonal anti-cA12Ab and were blocked with Tris-buffered saline (TBS)-Tween-10 % BSA. Plates were then incubated with duplicates of standard cA12 solutions, assay controls or mucosal extracts diluted in TBS-Tween-3 % BSA, after which they were incubated with horseradish peroxidase-labelled anti-cA12 polyclonal Ab. A 3,3’,5,5’-tetramethylbenzidine substrate was used for colour development and absorbance at 450 nm was measured. S100A12 concentrations in extracts of snap-frozen intestinal mucosal tissues were measured using the same lot of reagents for all samples and were reported as μg/L of the intestinal mucosal supernatant.

### Measurement of mucosal MPO activity

For MPO measurement, snap-frozen mucosal samples from the duodenum, jejunum, ileum and colon were weighed, suspended in 1 ml of ice-cold extraction buffer per 50 mg of tissue and homogenised for 2 × 50 s at 5000 × g using Precellys 24 ceramic beads at 4 °C (Bertin technologies, France). The extraction buffer contained hexadecyltrimethylammonium bromide (HTAB; 0.5 % weight/volume [w/v]) in a 50-mM sodium phosphate buffer (pH 5.4). After homogenisation, samples were centrifuged at 4000 × g and 4 °C for 20 min, and the supernatants were collected and stored at −80 °C. MPO activity was determined in intestinal mucosa using the method described by Marquez and Dunford [54] with some modifications. The final assay buffer was supplemented with HTAB as a cationic detergent. Briefly, the reaction mixture consisted of 170 μL of sodium phosphate buffer (80 mM, pH 5.4) with HTAB (0.5 % w/v) and 3,3’,5,5’-tetramethylbenzidine (1.6 mmol/L). Five μL of supernatant and 5 μL of distilled water were added to this reaction mixture, and the mixture was incubated while shaking at 37 °C for 6 min, then adding 20 μL of H_2_O_2_ (0.3 mmol/L). The final concentrations of the reaction mixture are reported in parentheses. After the addition of H_2_O_2_, kinetic measurement for 60 s was initiated at a wavelength of 620 nm using an automatic chemical analyser (Kone Pro, ThermoFisher Scientific, Vantaa, Finland). MPO activity was expressed as delta absorbance units per minute (ΔA/min).

The colorimetric method for MPO determination was validated for precision, accuracy and recovery. Intra- and inter-assay precisions were determined as coefficients of variation calculated from six different small and large intestinal mucosa samples within an analytical run (*n* = 10) and across different runs (*n* = 10). The linearity was evaluated using serial dilutions of the same mucosal samples (1:2, 1:4, 1:8 and 1:16) by calculating the mean and standard deviation (SD) of the observed and expected ratios (%). The spiking recovery was analysed among six different spiking activity samples of human MPO-pure enzyme (0.066, 0.109, 0.161, 0.191, 0.318 and 0.477 ΔA/min) in five different small and large intestinal mucosa samples. The mean and SD values were calculated for the observed and expected ratios (%).

### Statistical analysis

Data are presented as medians (interquartile range). The differences in the S100A12 concentrations and MPO activities between the four different segments of the intestine (duodenum, jejunum, ileum and colon) were analysed using an analysis of variance (ANOVA) model. To satisfy the assumptions of normality and the homogeneity of variances, the original values were transformed to a logarithmic scale. If the results of the Levene’s test were significant (*p* < 0.05) following data transformation, we used an ANOVA-Welch test for further analysis. The fitted model included the section of intestine as the fixed effect and the dog as the random effect. Tukey’s HSD test was used in pairwise comparisons to control for multiple comparisons, and we used the Tamhane’s T2 test for variables with significant differences in the variance. For all analyses, we considered values of *p* < 0.05 as significant. All statistical analyses were performed using the SAS 9.3 statistical software program (SAS Institute Inc., Cary, NC, USA).

## Abbreviations

ANOVA: Analysis of Variance
cA12: Canine S100A12 Protein
CCECAI: Canine Chronic Enteropathy Clinical Activity Index
CD: Crohn’s Disease
CV: Coefficients of Variation
EDTA: Ethylenediaminetetraacetic Acid
ELISA: Enzyme-Linked Immunosorbent Assay
HOCl: Hypochlorous Acid
HTAB: Hexadecyltrimethylammonium Bromide
IBD: Inflammatory Bowel Disease
MPO: Myelopreoxidase
O/E: Observed to Expected Ratios
PBS: Phosphate Buffered Saline
PCDAI: Paediatric Crohn’s Disease Activity Index
TBS: Tris Buffered Saline
SD: Standard Deviation
UC: Ulcerative Colitis
WSAVA: World Small Animal Veterinary Association
ΔA: Delta Absorbance
